# κ-opioid receptor agonist, U50488H, inhibits pyroptosis through NLRP3 via the Ca^2+^/CaMKII/CREB signaling pathway and improves synaptic plasticity in APP/PS1 mice

**DOI:** 10.3892/mmr.2021.12168

**Published:** 2021-05-25

**Authors:** Xiaofu Song, Zhiqiang Cui, Jiahuan He, Tuo Yang, Xiaohong Sun

**Affiliations:** 1 Department of Neurology, The Fourth Affiliated Hospital of China Medical University, Shenyang, Liaoning 110032, P.R. China; 2 Department of Neurology, The People's Hospital of Liaoning Province, Shenyang, Liaoning 110016, P.R. China

**Keywords:** κ-opioid receptor agonist, Alzheimer's disease, Ca^2+^/calcium/calmodulin-dependent protein kinase II/cyclic adenosine monophosphate-response element binding protein signaling pathway, pyroptosis, synaptic plasticity

## Abstract

Alzheimer's disease (AD) is a progressive neurodegenerative brain disorder with slow onset in most cases. Clinically, dementia associated with AD is characterized by memory disorders, aphasia, executive dysfunction and personality and behavior changes. Currently, treatment strategies attempt to reduce certain symptoms, however there is no cure for AD. The aim of the present study was to identify a novel treatment strategy for AD. Thus, the protective effects of a κ-opioid receptor (KOR) agonist, U50488H on neural damage in AD mice were investigated. The underlying mechanism of the Ca^2+^/calcium/calmodulin-dependent protein kinase II/cyclic adenosine monophosphate-response element binding protein (Ca^2+^/CaMKII/CREB) signaling pathway was evaluated. Amyloid precursor protein (APP)/presenilin-1 (PS1) mice were treated subcutaneously with a KOR agonist for 28 days. The learning and memory abilities of the APP/PS1 mice were evaluated using the Morris water maze test. Damage to hippocampal neurons was assessed using hematoxylin and eosin staining. Inflammatory factors and brain injury markers were detected using ELISA. Neurons were examined using immunofluorescence and dendritic spines were observed using Golgi-Cox staining. Western blotting was used to detect NOD-, LRR- and pyrin domain-containing protein 3, microglial ptosis and the Ca^2+^/CaMKII/CREB-related protein pathway. The KOR agonist significantly improved the brain injury observed in APP/PS1 mice, inhibited microglia pyroptosis and improved the synaptic plasticity of APP/PS1 mice, which was reversed by a KOR antagonist. Thus, the KOR agonist improved the symptoms of APP/PS1 mice by inhibiting the Ca^2+^/CaMKII/CREB signaling pathway.

## Introduction

Alzheimer's disease (AD) is a degenerative disease of the central nervous system (CNS) and is characterized by progressive memory loss and cognitive dysfunction (1). The neurological dysfunction observed in AD is accompanied by various cognitive symptoms and behavioral abnormalities that seriously affect the daily life and work of patients (1). The main pathological features of AD are amyloid plaques or senile plaques that are composed of extracellular β-amyloid protein (Aβ) and neurofibrillary tangles (NFT), formed by abnormal accumulation in cells of hyperphosphorylated tau protein (2). As the final manifestation of AD, neuron synaptic loss is primarily associated with the abnormal signal transmission of excitatory glutamate (Glu) and depolymerization of the cytoskeleton (3).

The N-methyl-D-aspartate receptors (NMDARs) are widely distributed on postsynaptic membranes of neurons in the CNS, particularly in the hippocampus (HC). NMDARs are essential signal mediators and play critical roles in synaptic transmission and synaptic plasticity (4,5). Over-activation of NMDARs leads to the opening of Ca^2+^ channels coupled with Glu receptors, resulting in increases in the intracellular concentration of Ca^2+^, which activates Ca^2+^/CaMKII (6). Phosphorylated (p) CaMKII directly activate cyclic adenosine monophosphate (cAMP) response element binding protein (CREB), a key regulator of long-term memory (7,8). p-CREB further regulates the synthesis of activity-regulated cytoskeleton-associated protein and synapsin, which participate in the formation of long-term-potentiation (LTP) and maintain the formation of long-term memory (9). Brain-derived neurotrophic factor is an essential target gene of CREB, which affects the formation of synapses and neuronal plasticity and plays a role in promoting the growth and development of neurons (10,11). Therefore, the Ca^2+^/CaMKII/CREB signaling pathway is closely interconnected with synaptic plasticity.

It is widely accepted that the inflammatory response induced by the activation of Aβ deposition may be the most important pathological mechanism of AD (12). Accumulating evidence indicates that, as the central element of the inflammatory response, the inflammasome is closely related to numerous immune inflammatory and metabolic diseases. Furthermore, it plays a vital role in the occurrence and development of nervous system diseases (13,14). Evidence suggests that Aβ and other abnormal aggregation proteins activate the inflammasome and promote the maturation and secretion of critical inflammatory factors, such as IL-1β that participate in the internal immune inflammatory response and cause pyroptosis, which is an essential natural immune response (15,16). Additionally, Aβ causes K^+^ to flow out of neurons and a low K^+^ concentration is an effective activator of the NOD-, LRR- and pyrin domain-containing protein 3 (NLRP3) inflammasome (17). The activated NLRP3 inflammasome participates in the neurotoxicity caused by pyroptosis, which aggravates neurodegenerative diseases and causes progressive cognitive impairment (18,19).

Microglia are important recruiters and executors of the inflammatory response in the brain (20,21). Once activated, microglia produce pro-inflammatory cytokines and other cytotoxic mediators, ultimately affecting the normal function of the brain (22). Previous studies have revealed that microglia may participate in the formation of synaptic connections between neurons and play a key role in the regulation of synaptic plasticity in the brain (23,24).

κ-opioid receptor (KOR) agonists are widely used in perioperative analgesia due to their strong analgesic effects; they also regulate emotional and cognitive functions (25). The KOR agonist, U50488H was found to reduce cognitive impairment significantly (26). Therefore, the present study investigated the effects of U50488H on spatial memory, synaptic plasticity and inflammatory cells in an amyloid precursor protein (APP)/presenilin-1 (PS1) mouse model. Synaptic function was assessed by examining the expression level of postsynaptic density protein 95 (PSD95). Furthermore, the underlying mechanism of microglia pyroptosis in AD and the regulatory mechanism of the Ca^2+^/CaMKII/CREB signaling pathway in synaptic plasticity were investigated. It was identified that the KOR agonist, U50488H regulated NLRP3 via the Ca^2+^/CaMKII/CREB signaling pathway, inhibiting microglial cell pyroptosis and improving synaptic plasticity in APP/PS1 mice.

## Materials and methods

### 

#### Animals and experimental groups

In total, 45 male (weight, 25–30 g; age, 6 months) AD mice expressing human APP and PS1 genes and 15 C57BL/6 mice (age, 6 months) were obtained from the Experimental Animal Center of the China Medical University. The present study was approved by the Animal Welfare and Ethics Committee of the China Medical University Laboratory (Institutional Animal Care and Use Committees approval no. 2018236). All mice were offered food and water *ad libitum* and housed in pathogen-free facilities under a 12-h light/dark cycle in a controlled room temperature of 12–24°C and 60% relative humidity. The mice used in the present study exhibited amyloid plaques and NFT from age 9–12 months. Upon completion of the experiments, mice were euthanized by intraperitoneal injection of 50 mg/kg body weight sodium pentobarbital, followed by heart perfusion or dislocation of the cervical spine. Brain tissue samples were collected from wild-type or APP/PS1 transgenic mice. The pathology was primarily localized to the HC, amygdala and cerebral cortex. To reduce potential variations among the experimental mice, the mice used in the present study were first grouped by similar weight and at 12-months old. Then mice from each litter were equally distributed to the different study groups. Specifically, the mice were randomly divided into four groups as follows: i) Wild-type mice (control group; n=15); ii) APP/PS1 transgenic mice (AD group; n=15); iii) APP/PS1 transgenic mice were treated with the κ-opioid receptor agonist, U50488H for 28 days (1.25 mg/kg) using an osmotic pump (U50488H group; n=15); iv) APP/PS1 transgenic mice were treated with U50488H for 28 days (1.25 mg/kg) using an osmotic pump and were injected through an intracerebral pump injection with CaMKII antagonist, KN93 (5 µM/day) for 28 days (KN93 group; n=15). KN93 (Sigma-Aldrich; Merck KGaA) was dissolved in 0.9% saline containing 1% DMSO and diluted to a concentration of 1 mM.

#### Morris water maze

Spatial memory was assessed using the Morris water maze test, which included the concealed platform test and the space exploration test (27,28). Before each trial period, the mice were brought to the room with the water maze to allow for acclimation. For spatial learning, the mice were trained for five consecutive days to find a hidden platform in the Morris water maze. During each trial, the mouse started from the middle of one of the four quadrants, facing the wall of the pool. The trial ended when the animal climbed onto the platform (diameter, 10 cm). The mice were not allowed to search for the platform for more than 60 sec, after which they were guided to the platform. For the space exploration test, the platform was removed after the end of the hidden platform test. Then, 24 h after the hidden platform test, the mice were placed in the same starting position as the hidden platform test and their swimming paths were recorded for 60 sec. The time each mouse spent in the original quadrant and the number of times each mouse crossed the original platform location were recorded. The Morris water maze video analysis system was used for data processing.

#### Immunohistochemistry

Upon completion of the water maze experiments, five mice from each group were anesthetized by intraperitoneal injection with 50 mg/kg sodium pentobarbital. Following anesthesia, mice were perfused through the heart with PBS and then with pre-cooled 4% (w/v) paraformaldehyde, then the mouse's brain was rapidly removed. The brain tissue was placed in an embedding box, 4% paraformaldehyde was added and the box was placed in a freezer at −80°C for 24–48 h for rapid freezing. A cold microtome was used to slice the tissue into 5-µm sections. The sections were deparaffinized and rehydrated through a graded series of alcohol. Hydrogen peroxide (3%) solution was used to inactivate endogenous peroxidases and the sections were washed once with PBS (pH 7.4). The sections were exposed to a 0.1 M sodium citrate solution for antigen retrieval. Next, the sections were incubated overnight at 4°C in Aβ antibody (cat. no. sc-28365; 1:1,000; Santa Cruz Biotechnology, Inc.). The next day the sections were washed three times in PBS, incubated with a biotin-labeled secondary antibody (cat. no. sc-525409; 1:1,000; Santa Cruz Biotechnology, Inc.) at 37°C for 30 min, then washed again in PBS. The 3,3′-diaminobenzidine, as a chromogen, was used to stain sections for 3 min at room temperature to visualize the Aβ-positive cell staining. The cell nuclei were counterstained with hematoxylin, a neutral resin was used to seal coverslips onto the microscope slides and the stained sections were observed with a light microscope (Olympus Corporation) with a magnification of ×40.

#### Hematoxylin and eosin (H&E) staining

The sections that were mounted on glass microscope slides were heated to melt the paraffin. Then the sections were deparaffinized and rehydrated through a series of graded alcohol. The tissue sections were rinsed with tap water for 10 min. Hematoxylin staining was performed for 2 min at room temperature and the sections were rinsed with tap water for 10 min and immersed in 1% hydrochloric alcohol for 3 sec. Eosin staining was performed for 1 min at room temperature, then 95% ethanol followed by two changes of anhydrous ethanol were used to dehydrate the sections. The sections then were immersed in two changes of xylene for 5 min each. Finally, a neutral mounting medium was used to seal coverslips over the sections. Pathological changes in the tissue sections were observed using a light microscope (Olympus Corporation) with a magnification of ×40.

#### ELISA assay

At the end of the water maze experiment, five mice in each group were sacrificed by dislocating the cervical spine. The brain of the mice was collected. The brain tissue was lysed in Laemmli buffer with homogenization and sonication three time for 10 sec/time on ice (QSonica LLC). Following tissue homogenization, the Glu (cat. no. CES122Ge; Wuhan USCN Business Co., Ltd.), collagen II (cat. no. CB85527920; Boswio; http://www.boswio.com), IL-18 (cat. no. SEA064Mu; Wuhan USCN Business Co., Ltd.) and IL-1β (cat. no. SEA563Mu; Wuhan USCN Business Co., Ltd.) content was detected using ELISA kits according to the manufacturer's instructions. The kit was equilibrated to room temperature and the required reaction plate was removed. Then the standards and diluted samples were added into the wells of the corresponding reaction plate and incubated at room temperature for 20 min. Subsequently, the reaction plate was washed, HRP-labeled secondary antibody provided in the kit was added to each well and incubated at 37°C for 30 min. The plate was rinsed, and the color developing solution was added in the dark. The plate was incubated for 15 min at room temperature, and then the stop solution was added to each well. The optical density (OD) value at 450 nm was read using a microplate reader. The OD value was used as the vertical coordinate and the standard concentrations were used as the horizontal coordinate. A standard curve was constructed and the curve equation and R-value were calculated to determine the corresponding concentration values for each sample.

#### Golgi-Cox staining

The brain tissue samples were immersed in a 30% sucrose solution overnight followed by immersion in optimal cutting temperature embedding reagent at 4°C for 6 h, then stored at −80°C for 24 h and sectioned (thickness, 100 µm), according to a previous publication (27). The Golgi-Cox staining procedure was performed according to the instructions from the FD Rapid GolgiStain™ kit (FD Neurotechnologies, Inc.). The dendritic spine density of neurons was analyzed using ImageJ software (version 1.52a; National Institutes of Health).

#### Western blotting

The brain tissue samples were homogenized in iced-cold RIPA buffer containing protease inhibitor (Santa Cruz Biotechnology, Inc.) and protein concentration was estimated using BCA reagent. Then, 30 mg protein lysates per lane were loaded, separated by 6–12% SDS-PAGE gradient gels and transferred onto nitrocellulose membranes, then the membranes were blocked in a 5% skimmed milk solution at room temperature for 1 h. Tris-buffered saline with 0.1% Tween (TBST) was used to wash the membranes and then the membranes were incubated with the primary antibody including anti-NMDAR (1:1,000; cat. no. ab274377; Abcam), anti-p-CaMKII [1:1,000; cat. no. 12716S; Cell Signaling Technology, Inc. (CST)], anti-CaMKII (1:1,000; cat. no. 3362S; CST), anti-p-CREB (1:1,000; cat. no. 9198S; CST), anti-CREB (1:1,000; cat. no. 9197S; CST), anti-NCAM (1:1,000; cat. no. 99746S; CST), anti-NR2B (1:1,000; cat. no. ab254356; Abcam), anti-GluR1 (1:1,000; ab183797; Abcam), anti-SYN (1:1,000; cat. no. ab212184; Abcam), anti-PSD95 (1:1,000; cat. no. 3409S; CST), anti-GAPDH (1:1,000; cat. no. 2118S; CST), anti-pro-caspase-1 (1:1,000; cat. no. 24232S; CST), anti-pro-IL-1β (1:1,000; cat. no. 31202S; CST), anti-ASC (1:1,000; cat. no. 67824S; CST) and NLRP3 (1:1,000; cat. no. 15101S; CST) overnight at 4°C. The PVDF membranes were washed three times in PBST, incubated with anti-rabbit IgG, HRP-linked antibody (1:1,000; cat. no. 7074S; CST) at room temperature for 1 h and the PVDF membranes were washed in TBST. The labeled protein bands were visualized using an ECL kit (Amersham Biosciences) according to the manufacturer's instructions.

#### Immunofluorescence

Paraffin-embedded tissues were sectioned (thickness, 4 µm) using a microtome and placed on glass microscope slides. The sections were deparaffinized at room temperature by immersion in two changes of xylene and two changes of absolute ethanol for 10 min each. Subsequently, the sections were rehydrated in a graded series of ethanol and washed in 10 mM PBS three times for 5 min each. Antigen retrieval was performed at 95°C for 3 min. After the sections were cooled to room temperature, they were washed in PBS, immersed in 5% normal goat serum (Invitrogen; Thermo Fisher Scientific, Inc.) in PBS and maintained at 37°C for 1 h. Sections were then incubated with anti-ionized calcium binding adaptor molecule 1 (IBA1; 1:100; cat. no. sc-32725; Santa Cruz Biotechnology) and NLRP3 (1:50; cat. no. NBP2-12446; Novus Biologicals, LLC) or anti-ASC (1:800; cat. no. 67824S; CST) and anti-pro-caspase-1 (1:100; cat. no. NBP2-15713; Novus Biologicals, LLC) antibodies at 4°C overnight. The next day, the sections were washed with PBS and incubated with goat anti-rabbit IgG antibody (1:1,000; cat. no. 8889S; CST) or goat anti-mouse IgG antibody (1:1,000; cat. no. 4408S; CST) at 37°C for 1 h, then washed with PBS. DAPI was added at room temperature for 7 min. Subsequently, the sections were sealed with neutral mounting medium and observed with a fluorescence microscope.

#### Statistical analysis

The data were analyzed using SPSS version 21.0 (IBM Corp.) and are presented as means ± standard deviation. Differences between two groups were analyzed using unpaired Student's t-test. Differences among multiple groups were analyzed using one-way ANOVA followed by Tukey's post hoc test for pairwise comparisons. P<0.05 was considered to indicate a statistically significant difference.

## Results

### 

#### KOR agonist, U50488H, diminishes brain injury in APP/PS1 mice

**Figure 1.**
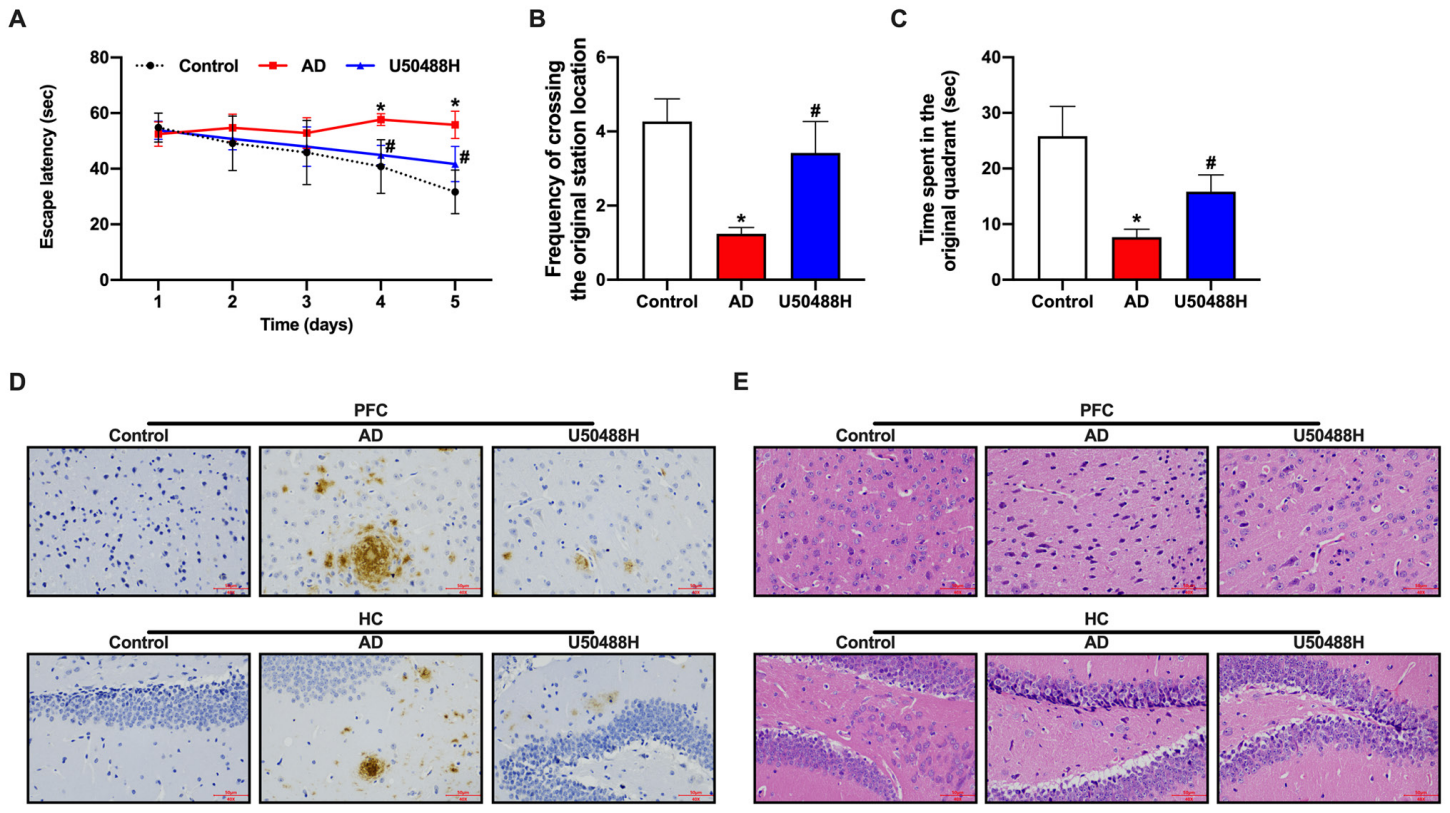
KOR agonist U50488H improves brain injury in amyloid precursor protein/presenilin-1 mice. (A) The escape latency of mice in each group during the Morris water maze test. (B) Frequency of crossing the original station location. (C) Time spent in the original quadrant. (D) Immunohistochemistry of brain tissue samples. The arrows indicate the positive cells. Scale bar, 50 µm. (E) Hematoxylin and eosin staining of brain tissue samples. Scale bar, 50 µm. *P<0.05 vs. Control group and ^#^P<0.05 vs. AD group. Experiments were performed in triplicate and repeated three times. KOR, κ-opioid receptor; AD, Alzheimer's disease; PFC, prefrontal cortex; HC, hippocampus.

To investigate the effect of the KOR agonist, U50488H, on the spatial memory of APP/PS1 mice, U50488H was administered subcutaneously for 28 days, using an osmotic pump. The Morris water maze was used to evaluate the cognitive function of the mice. Although impaired learning and spatial memory in the Morris water maze was observed in APP/PS1 mice compared with control mice, the mice in the U50488H group showed a significant improvement in their cognitive abilities (Fig. 1A-C). Aβ plaque deposition was decreased in the prefrontal cortex and HC in the APP/PS1 mice treated with U50488H (Fig. 1D). The pathological changes in brain tissue samples were observed using H&E staining. The neurons in 12 month-old APP/PS1 mice were sparse and disorganized, with the loss of numerous neurons. However, the morphology of neurons in the group treated with U50488H was improved (Fig. 1E). Furthermore, the expression of PSD95 was increased in the HC of U50488H-treated mice. Taken together, these results demonstrated that U50488H reduced brain injury in APP/PS1 mice.

#### KOR agonist, U50488H inhibits Ca^2+^ overload and improves synaptic plasticity in APP/PS1 mice

**Figure 2.**
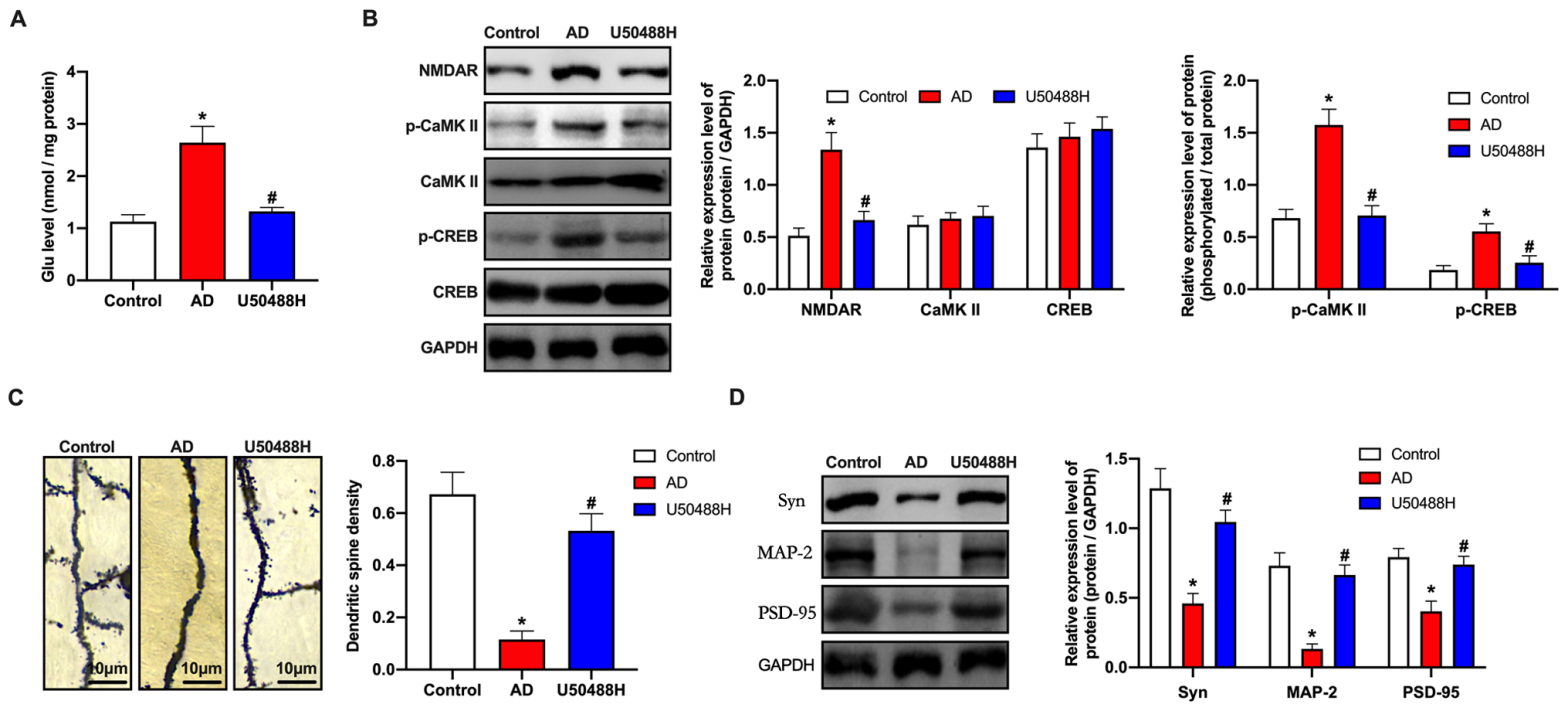
KOR agonist inhibits Ca^2+^ overload and improves synaptic plasticity in amyloid precursor protein/presenilin-1 mice. (A) ELISA analysis demonstrated the Glu concentration of mice in each group. (B) Western blot analysis of NMDAR, CaMKII, p-CaMKII, CREB and p-CREB expression levels of mice in each group. (C) Golgi-Cox staining of brain tissue samples. Scale bar, 10 µm. (D) Western blot analysis demonstrating NCAM, NR2B, GluR1, PSD95 and SYN expression levels of mice in each group. *P<0.05 vs. Control group; ^#^P<0.05 vs. AD group. Experiments were performed in triplicate and repeated three times. KOR, κ-opioid receptor; Glu, glutamate; NMDAR, N-methyl-D-aspartate receptor; CaMKII, calcium/calmodulin-dependent protein kinase II; p, phosphorylated; CREB, cyclic adenosine monophosphate response element binding protein; NCAM, neural cell adhesion molecule; NR2B, N-methyl D-aspartate receptor subtype 2B; GluR1, glutamate receptor 1; PSD95, postsynaptic density protein 95; SYN, α-synuclein; AD, Alzheimer's disease.

Glu is the primary neurotransmitter in the CNS. When the Glu concentration increases significantly, extensive pathological damage occurs in brain tissues (29,30). ELISA analysis revealed that Glu concentrations in the APP/PS1 mice were significantly higher than in the U50488H treatment group (Fig. 2A). As the Ca^2+^/CaMKII/CREB signaling pathway is involved in synaptic plasticity, the central components of the pathway, including NMDAR, CaMKII, p-CaMKII, CREB and p-CREB were analyzed. Based on the western blot results, the expression levels for NMDARs and the phosphorylation levels of CaMKII and CREB in the U50488H-treated group decreased significantly when compared to the APP/PS1 mice (Fig. 2B). Golgi-Cox staining was used to analyze the number of dendritic spines in the HC. The results demonstrated that the number of spines increased in the treated mice when compared to the APP/PS1 mice, which indicated that U50488H helped improve the growth of dendritic spines and improved synaptic plasticity in the treated mice (Fig. 2C). To investigate the underlying mechanism, western blot analysis was performed to detect the synaptic plasticity-related proteins, neural cell adhesion molecule, N-methyl D-aspartate receptor subtype 2B, Glu receptor 1, PSD95 and α-synuclein. The expression levels for these proteins were observed to be higher in the U50488H-treated group than in the APP/PS1 mice (Fig. 2D). These results demonstrated that treatment with U50488H improved synaptic plasticity in APP/PS1 mice.

#### KOR agonist, U50488H inhibits microglial pyroptosis in APP/PS1 mice

**Figure 3.**
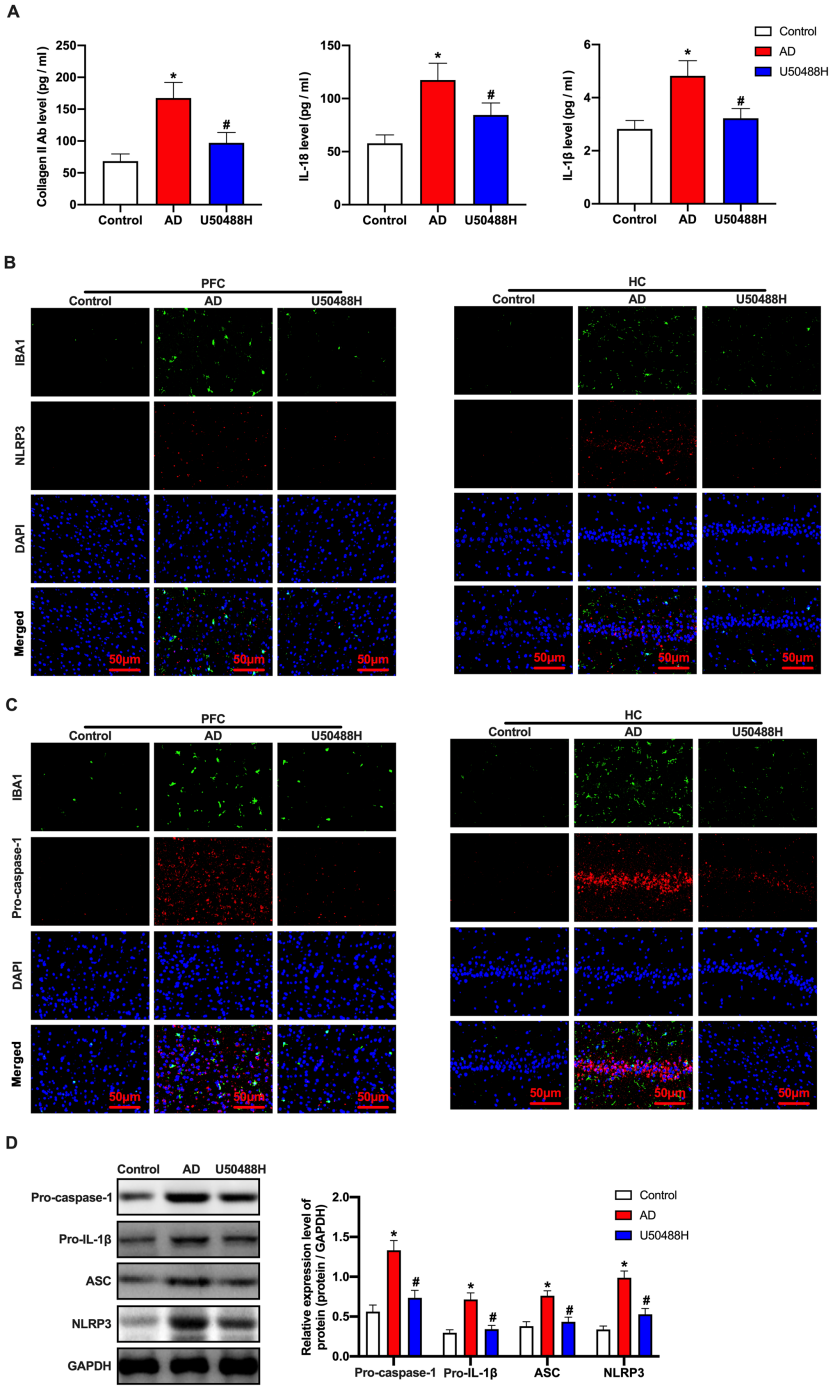
KOR agonist inhibits microglial pyrolysis in amyloid precursor protein/presenilin-1 mice. (A) The level of collagen II antibody, IL-18 and IL-1β were detected by ELISA. (B) The NLRP3 and IBA1 expression levels and (C) pro-caspase-1 and IBA1 expression levels of mice in each group were detected by immunofluorescence. Scale bars, 50 µm. (D) Western blot analysis of ASC, pro-IL-1β, pro-caspase-1 and NLRP3 expression levels of mice in each group. *P<0.05 vs. Control group; ^#^P<0.05 vs. AD group. Experiments were performed in triplicate and repeated three times. KOR, κ-opioid receptor; Glu, glutamate; NLRP3, NOD-, LRR- and pyrin domain-containing protein 3; IBA1, ionized calcium binding adaptor molecule 1; ASC, apoptosis-associated speck-like protein containing a C-terminal caspase recruitment domain; AD, Alzheimer's disease.

It has previously been reported that cell pyroptosis depends on cysteine aspartic acid-specific protease to promote the release of the inflammatory mediators, IL-1β and IL-18 (31). Notably, the levels of collagen II antibody, IL-18 and IL-1β in the serum of mice treated with U50488H decreased significantly (Fig. 3A). This indicated that U50488H played a critical role in inhibiting microglial pyroptosis in APP/PS1 mice. Therefore, immunofluorescence was performed to detect cell pyroptosis-related proteins. The results showed that the expression levels of NLRP3 and pro-caspase-1 in the U50488H-treated mice decreased significantly (Fig. 3B and C). To confirm the fluorescence results, pro-caspase-1, pro-IL-1β, apoptosis-associated speck-like protein containing a C-terminal caspase recruitment domain (ASC) and NLRP3 were analyzed using western blot analysis. The expression levels of ASC, pro-IL-1β, pro-caspase-1 and NLRP3 in the U50488H-treated group were significantly lower when compared with those of the APP/PS1 mice (Fig. 3D). These results indicated that the KOR agonist inhibited microglial pyroptosis in APP/PS1 mice.

#### CaMKII inhibitor, KN93 blocks changes in synaptic plasticity in APP/PS1 mice induced by the KOR agonist

**Figure 4.**
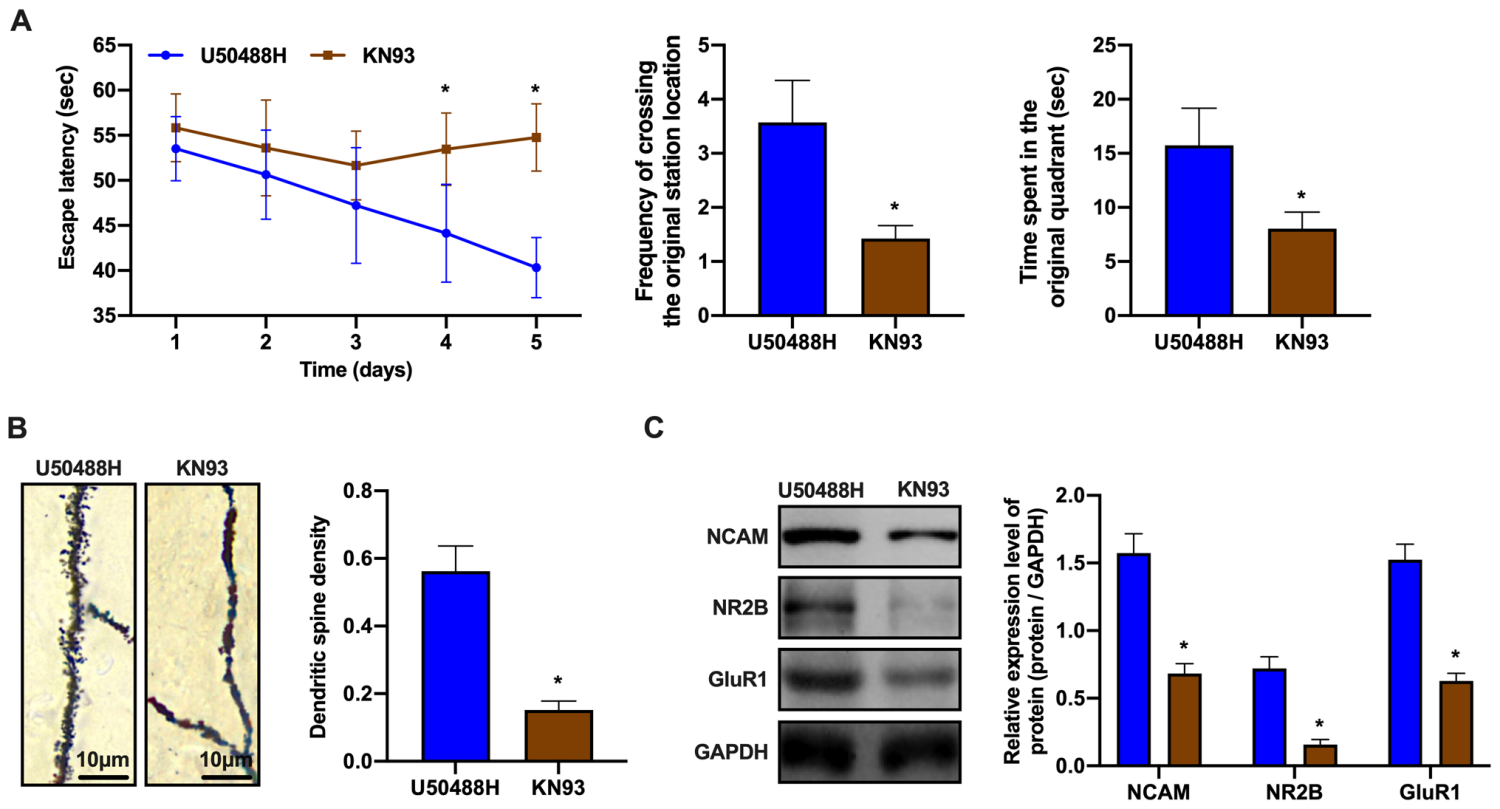
CaMKII inhibitor, KN93 blocks the KOR agonist-induced changes of synaptic plasticity of amyloid precursor protein/presenilin-1 mice. (A) The escape latency, the frequency of crossing the original station location and the time spent in the original quadrant of mice in each group. (B) Golgi-Cox staining of brain tissue samples. Scale bar, 10 µm. (C) Western blot analysis of NCAM, NR2B and GluR1 expression levels of mice in each group. *P<0.05 vs. U50488H group. Experiments were performed in triplicate and repeated three times. CaMKII, calcium/calmodulin-dependent protein kinase II; KOR, κ-opioid receptor; NCAM, neural cell adhesion molecule; NR2B, N-methyl D-aspartate receptor subtype 2B; GluR1, glutamate receptor 1.

CaMKII is an important member of the calmodulin regulatory protein family and plays a role in the pathophysiological processes of numerous diseases, such as neuropsychological disorders (32). Therefore, the regulatory mechanisms of the Ca^2+^/CaMKII/CREB signaling pathway in synaptic plasticity were investigated. KN93 was injected intraperitoneally into APP/PS1 mice for 28 days. The mice also received U50488H, which was administered subcutaneously for 28 days using an osmotic pump. KN93 is a CaMKII specific inhibitor, which inhibits its phosphorylation activity. Learning and memory was assessed in the treated and control mice using the Morris water maze. The learning and memory abilities of the KN93-treated group was observed to decrease (Fig. 4A). In addition, the density of the dendritic spines in the CA1 area of the HC was evaluated using Golgi-Cox staining. The dendritic spine density in the KN93-treated group was decreased when compared to that of the untreated group (Fig. 4B). Furthermore, the mechanisms by which the CaMKII antagonist blocked changes in synaptic plasticity induced by the KOR agonist were evaluated. Western blot analysis showed that the expression of synaptic plasticity-related proteins decreased in the KN93-treated group (Fig. 4C). These observations demonstrated that the CaMKII inhibitor blocked the effects of U50488H, which had improved synaptic plasticity in the APP/PS1 mice.

#### CaMKII inhibitor eliminates inhibition by the KOR agonist on microglial pyroptosis in APP/PS1 mice

**Figure 5.**
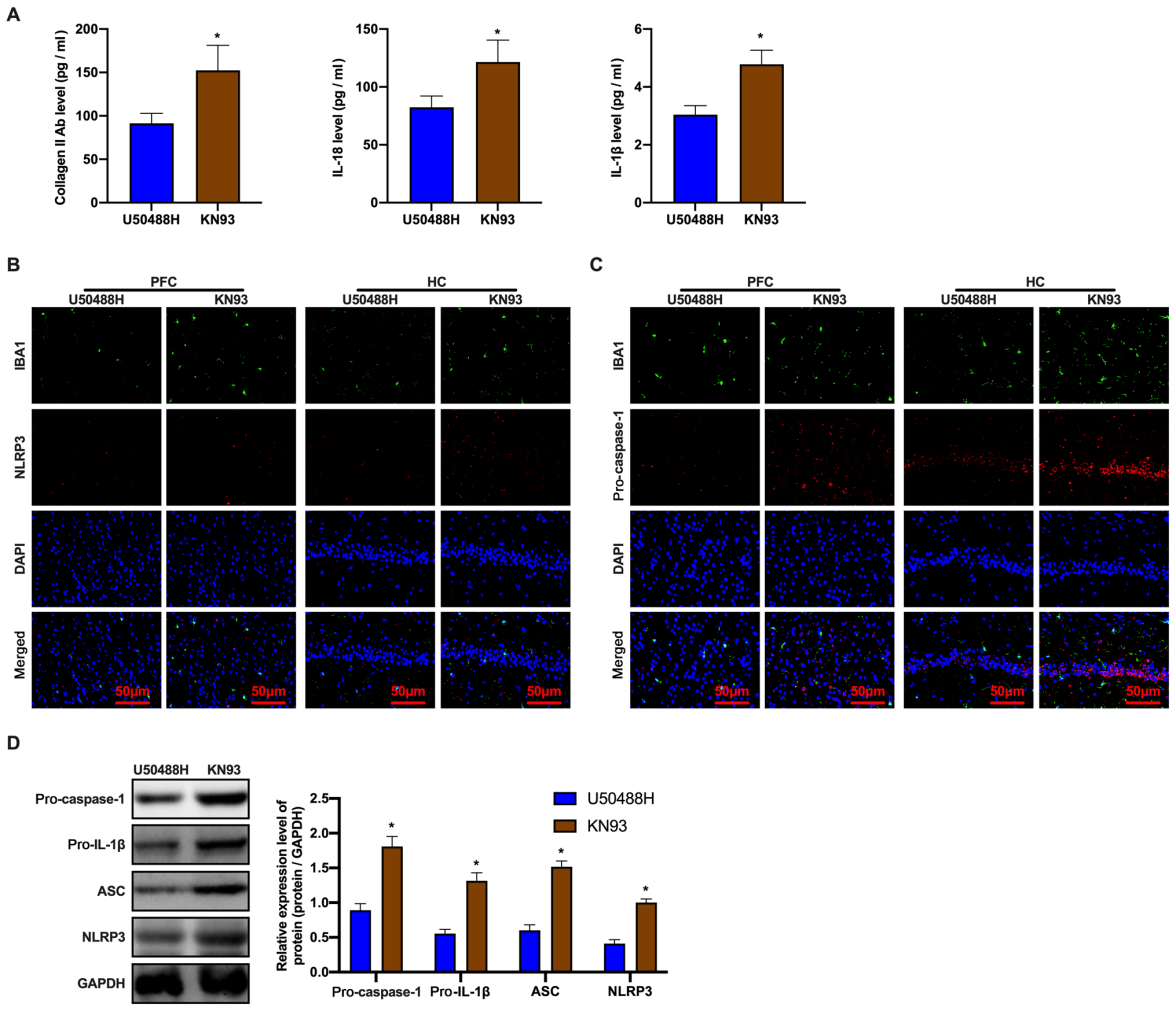
CaMKII inhibitor, KN93 eliminates the inhibition of KOR-agonist, U50488H on pyroptosis of amyloid precursor protein/presenilin-1 mice microglia. (A) The level of collagen II antibody, IL-18 and IL-1β were detected by ELISA. (B) The NLRP3 and IBA1 expression levels and (C) The pro-caspase-1 and IBA1 expression levels of mice in each group detected by immunofluorescence. Scale bar, 50 µm. (D) Western blot analysis of ASC, pro-IL-1β, pro-caspase-1 and NLRP3 expression levels of mice in each group. *P<0.05 vs. U50488H group. Experiments were performed in triplicate and repeated three times. CaMKII, calcium/calmodulin-dependent protein kinase II; KOR, κ-opioid receptor; NLRP3, NOD-, LRR- and pyrin domain-containing protein 3; IBA1, ionized calcium binding adaptor molecule 1; ASC, apoptosis-associated speck-like protein containing a C-terminal caspase recruitment domain; PFC, prefrontal cortex; HC, hippocampus.

Based on the abovementioned results, it was hypothesized that the CaMKII inhibitor blocked inhibition of the KOR agonist on pyroptosis. The ELISA assay revealed a significant increase in the expression levels of pyroptosis-related proteins (Fig. 5A). Treatment with KN93 prevented the decrease in NLRP3 and pro-caspase-1 protein expression when compared to mice in the U50488H-treated group (Fig. 5B and C). In addition, changes in the expression of pyroptosis-related proteins were examined. The results consistently demonstrated that the expression levels of NLRP3, ASC, pro-caspase-1 and pro-IL-1β proteins increased in the KN93-treated mice (Fig. 5D). These observations demonstrated that the inhibition of pyroptosis in APP/PS1 mice microglia by the KOR agonist could be eliminated by KN93 treatment.

## Discussion

Opioid receptors are widely but unevenly distributed in the nervous system (33). There are at least four opioid receptor subtypes in the CNS, µ, κ, δ and σ. The present study focused on KORs. In addition to the dentate gyrus, KORs are expressed in the hypothalamus, cerebral cortex and spinal cord (34). Opioids are common analgesic treatments in the clinical setting and KOR agonists exert similar effects. Moreover, compared with traditional opioids, KOR agonists antagonize the effects mediated by µ-opioid receptors in the brain, including memory processes. Prior studies have demonstrated that KOR-specific agonists exhibit antinociceptive effects and, unlike morphine and other opioid analgesics, KOR agonists do not result in respiratory depression or addictive effects (35). However, KORs agonists in the CNS produce irritable and sedative effects (36). U50488H is a KOR agonist. Acetylcholine is blocked through the KORs in the nervous system, which blocks the decrease in acetylcholine release and ultimately improves learning and memory (37). However, the specific mechanism by which KOR agonists promote recovery from brain injury in AD remains unclear. In the present study, the Ca^2+^/CaMKII/CREB signaling pathway was found to play an essential role in this process.

The Ca^2+^/CaMKII/CREB signaling pathway is a critical signal transduction pathway and is involved in the formation and maintenance of learning and memory in the CNS (38,39). Ca^2+^ is a second messenger that participates in a range of physiological and biochemical processes in cells. In the process of cell signal transduction, CaM is the receptor for Ca^2+^, forming the Ca^2+^/CaM complex. As an important target enzyme for Ca^2+^, CaMKII phosphorylates numerous substrates to participate in neuronal plasticity, synthesis, neurotransmitter release and LTP (40). cAMP regulatory element modulator can be phosphorylated by CaMKII, which regulates gene transcription and enhances LTP formation in the HC (41). The Ca^2+^/CaMKII/CREB signaling pathway also regulates synaptic plasticity, which is consistent with the experimental results of the present study. The KOR agonist inhibited Ca^2+^ overload and improved synaptic plasticity in APP/PS1 mice in the present study and the CaMKII inhibitor blocked those changes.

In the present study, it was shown that the Ca^2+^/CaMKII/CREB signaling pathway could regulate microglial pyroptosis. The KOR agonist significantly inhibited the inflammatory response according to the ELISA analysis. In addition, the immunofluorescence results that detected cell pyroptosis-related proteins revealed that the KOR agonist inhibited microglial pyroptosis in APP/PS1 mice. It has been established that KOR agonists significantly improved cognitive dysfunction in cardiopulmonary bypass rats via the JAK2/STAT3 signaling pathway (42). However, to the best of our knowledge, it has not previously been reported that KOR agonists could improve memory impairment in AD. In the present study, U50488H regulated synaptic plasticity and microglia through the Ca^2+^/CaMKII/CREB signaling pathway and the CaMKII inhibitor reversed this outcome. These observations provide theoretical evidence that might prove useful for future treatment of patients with AD.

Thus, the present study demonstrated that KOR agonists provided neuroprotective effects against AD brain damage in APP/PS1 mice, which was at least partially mediated by inhibition of the Ca^2+^/CaMKII/CREB signaling pathway. Further investigation is required to assess the possible associations among other signaling pathways involved in the underlying mechanisms by which KOR agonists are able to repair the damage that occurs in the AD brain.

## Data Availability

The datasets used and/or analyzed during the current study are available from the corresponding author on reasonable request.
